# The impact of social media on body image and weight-change behaviors among young people: a systematic scoping review

**DOI:** 10.1186/s40337-026-01717-4

**Published:** 2026-08-08

**Authors:** Jiayi Zhou, Lijuan Xu, Caifu Li, Zhirun Wang

**Affiliations:** Medicine School of Lishui University, Lishui University, Lishui, Zhejiang Province China

**Keywords:** Social media, Young people, Body image, Weight-change behaviors, Appearance comparison, Disordered eating

## Abstract

**Background:**

Young people aged 10–24 years are the core users of social media (SM), and are particularly vulnerable to idealized body standards propagated on these platforms. While social media can promote healthy lifestyles, and substantial evidence has confirmed its significant association with body dissatisfaction and unhealthy weight-change behaviors among young people, how specific social media content characteristics shape the full spectrum of weight-change behaviors through differentiated mechanisms remains incompletely understood. Existing evidence, while consistent on core associations, is fragmented regarding causal pathways, content-specific effects, and cross-cultural generalizability. This scoping review aimed to synthesize current empirical evidence on the impact of social media on body image and weight-change behaviors among young people.

**Method:**

This review was conducted in line with the Preferred Reporting Items for Systematic Reviews and Meta-Analyses extension for Scoping Reviews (PRISMA-ScR) guidelines. A systematic literature search was performed across PubMed, Scopus, Web of Science, Embase, and the Cochrane Library. Studies were included based on a pre-defined PIO framework, and methodological quality of included studies was assessed descriptively using validated tools.

**Results:**

SM was found to exert a significant impact on young people’s BI and weight-change behaviors. SM platforms propagate idealized body standards (leanness for females, muscularity for males), which are closely linked to BD, restrictive eating, excessive exercise, and misuse of weight-loss products, with unscientific weight-management information being widely disseminated on such platforms. Notably, the type of SM content had a more pronounced impact on young people than usage duration, and gender-specific differences in weight-change behaviors were identified. Although a small amount of anti-diet and body-positive content on SM exhibited limited protective effects against negative BI, such effects were overshadowed by the preponderance of negative content. These findings highlight the urgent need to promote diverse body representations on SM, regulate harmful content, and enhance young people’s digital literacy.

**Conclusions:**

SM exerts a predominantly negative impact on the weight-change behaviors of young people, with significant effects on BI perception. SM platforms, content creators, and healthcare professionals should collaborate to promote body diversity and evidence-based health content, while enhancing young people’s digital literacy to mitigate the adverse effects of SM. These measures are critical to ensuring healthy weight management in this developmentally vulnerable population.

**Supplementary Information:**

The online version contains supplementary material available at https://doi.org/10.1186/s40337-026-01717-4.

## Abstract (plain language summary)

Social media (SM) is an integral part of daily life for young people aged 10–24 years, and exerts a strong influence on their body image perception and weight-related decisions. Most content on SM platforms features unrealistic perfect body types (e.g., extreme leanness or muscularity), which leads many young people to feel dissatisfied with their own bodies and adopt unhealthy habits such as extreme dieting, over-exercising, or using unproven weight-loss products. While anti-diet and body-positive content on SM can slightly improve young people’s body satisfaction, these positive effects are negligible compared with the negative impact of mainstream SM content. To make SM a safer space for young people, it is essential to teach them to think critically about SM content, promote diverse body types online, and share evidence-based health information about weight management. Healthcare experts, SM platforms, and content creators all play a key role in supporting the physical and mental well-being of young people.

**Supplementary Information:**

The online version contains supplementary material available at https://doi.org/10.1186/s40337-026-01717-4.

## Background

From a contemporary biopsychosocial perspective, adolescence is defined as a developmental stage spanning 10–24 years, representing a critical transition from puberty to the assumption of adult social roles [1]. This demographic accounts for 24% of the global population [2] and is characterized by profound physiological, psychological, and emotional transformations [3], alongside heightened sensitivity to social feedback and inherent vulnerability to external cognitive inputs—factors that can impair emotional regulation capabilities [3, 4]. Against the backdrop of developmental physical and emotional susceptibility, social media has become an integral part of young people’ s daily lives as they undergo key life transitions: schooling and graduation, family separation, and transition from education to employment [5]. As the core user cohort of social media (SM), these developmental traits render young people particularly susceptible to the idealized body standards propagated on digital platforms.

In the digital era, SM and associated digital tools (e.g., smartphones, social networking applications) have become an indispensable component of young people’ s daily lives [6]. As of February 2025, approximately 64% of the global population uses SM, with 25% of these users primarily following celebrities and internet influencers through SM platforms [7]. SM plays a pivotal role in shaping personal identity, influencing individual perspectives, and facilitating the formation of behavioral motivations [8]; while it can actively promote healthy lifestyles, SM also induces a range of negative psychological and behavioral outcomes in young people. Within Western cultural contexts, overweight and obesity are often perceived as unfavorable traits, whereas a slender physique is widely regarded as an attractive attribute [9]. Unlike traditional media, SM enables real-time physical appearance comparison among users and encourages user-generated content creation, which further stimulates and reinforces the pursuit of idealized physical appearance [10].

Body Image (BI) is a multidimensional construct encompassing individuals’ perceptions, cognitions, emotions, and attitudes toward their own bodies, with a specific focus on physical appearance, body shape, and bodily functions [11]. Appearance-centered SM platforms are known to shape young people’s relationship with their own bodies [12], significantly increase body shame [13], and contribute to negative BI outcomes such as BD [14]. Driven by the desire to conform to SM-promoted ideal body shapes, young people are prone to developing unhealthy eating habits [15], including strict dietary control, the moralization of so-called healthy eating [16], and inappropriate weight-loss attempts [17, 18]. Weight misperception and negative attitudes toward body weight further drive weight-control behaviors in young people, which are associated with increased psychological and social risks of disordered eating (DE) and chronic disease [19, 20]. Unhealthy weight control behaviors (UWCBs) refer to a range of maladaptive behaviors used for weight loss, gain or maintenance, including but not limited to fasting, excessive exercise, caloric restriction, self-induced vomiting, and use of weight-loss medications [21]. Epidemiological evidence has confirmed a high prevalence of UWCBs among female high school students, a trend that is significantly correlated with SM usage, distorted weight perception, and weight-related teasing [21].

As platforms like Instagram and Snapchat continue to influence societal aesthetic standards, understanding their impact on body image dissatisfaction has become increasingly crucial [22]. Several studies have summarized the relationship between social media usage and body image or eating disorders [10, 14, 23, 24].These works consistently show that SM use is associated with body dissatisfaction (BD) and disordered eating, with social comparison, thin-ideal internalization, and self-objectification as key mediators.Nevertheless, most existing reviews have focused predominantly on the symptomatic manifestations of clinical eating disorders or general body dissatisfaction, rather than the broader spectrum of weight-change behaviors—encompassing both healthy and unhealthy attempts to lose, gain, or maintain body weight. UWCBs are highly prevalent among young people yet have received limited systematic attention in the review literature. Furthermore, the rapid evolution of social media platforms and the emergence of novel content trends necessitate an updated and comprehensive synthesis of the available evidence.Notably, the bulk of this evidence originates from high‑income Western settings, with a conspicuous lack of studies conducted in low‑ and middle‑income countries (LMICs), which limits the cross‑cultural applicability of current findings.

Therefore, the main objective of the present scoping review is to map and describe how exposure to social media content has been investigated in relation to body image perception and weight-change behaviors among young people aged 10–24 years.

## Method

### Research question

The review is guided by the following research question: How does social media affect the body image and weight-change behaviors of young people? The research was conducted by a team of two scholars and aimed to summarize the current status of studies in the literature regarding the relationships among social network usage by young people, body image, and weight-change behaviors. This study used the scoping review method to conduct a comprehensive search of the literature on the five-step process proposed by Arksey and O’Malley [25]. The specific steps of this method include (i) determining the research question, (ii) screening relevant research literature, (iii) study selection, (iv) data organization, and (v) result summarization and reporting [25].

### Search strategy

To screen relevant studies from September to October 2025, we developed a search strategy using MeSH terms, keywords, and free text combined with Boolean operators (AND/OR/quotes/parentheses). The specific search terms are detailed in Table S1. Literature searches were conducted in the PubMed, Scopus, Web of Science (WOS), Embase, and Cochrane Library databases, ultimately yielding 1,717 articles. The literature search process strictly followed the standard procedures of the Preferred Reporting Items for Systematic Reviews and Meta-Analyses (PRISMA) extension for scoping reviews (PRISMA-ScR) [26].

### Study eligibility criteria

The review was guided by the participants, interventions, and outcomes (PIO) questions.

Participants (P): Young people aged 10–24 years.

Intervention or exposure (I): The included studies must analyze visual, narrative, or symbolic content related to body image and weight-change behaviors posted by social media users and institutional accounts, such as posts, pictures, topic tags, and comments. Included studies were published between 2016 and 2025 and written in English.

The exclusion criteria include the following two types of studies First, no analysis of spontaneous social media content related to body image or weight.

- The study subjects are young people, but social media is used only as a content dissemination channel, and the visual or symbolic content of social media is not analyzed.
- The study is an intervention aimed at improving mental health or nutritional status but does not assess the influence of spontaneously occurring content on social media (i.e., content that is naturally present rather than introduced by the researchers).
- The study is a project conducted on platforms such as Facebook or Twitter, where the research focus is solely on the effect of the project itself, without examining users’ free interactions with aesthetic or social-norm content.Second, studies that focus on traditional media, such as newspapers, radio, and television.

Systematic reviews and meta-analyses were eligible for inclusion in the present scoping review if they synthesized primary studies that would otherwise have met the PIO criteria individually. This approach is consistent with scoping review methodology, which permits the inclusion of secondary syntheses to map the breadth and nature of existing evidence at a higher level [25, 27]. To avoid double-counting of primary findings, results from included systematic reviews were extracted and reported distinctly at the level of the synthesis (i.e., the review’s own conclusions, design features, and identified gaps) and were not disaggregated into individual primary study results for the purposes of effect-size or outcome reporting. Potential overlap in underlying primary literature was documented narratively, and the study characteristics table (Table 1) clearly identifies which records are systematic reviews, enabling the reader to evaluate the degree of conceptual overlap.

Outcomes (O): We included studies that focused on the impact of young people’s social media content on their self-image or body perception as the core evaluation metric and specifically examined the relevant results involving changes in weight management strategies.

## Study selection

In the first round of screening, two reviewers (J Zhou and Z Wang) independently conducted a preliminary assessment of the titles and abstracts of all the retrieved literature. If a document contained only the title but not the abstract, it was directly included in the subsequent data organization stage for further review during the full-text assessment phase.Discrepancies arising at any stage were resolved through discussion to reach a consensus; if consensus could not be achieved, a third reviewer (L Xu) was consulted to make the final decision.

**Fig. 1:**
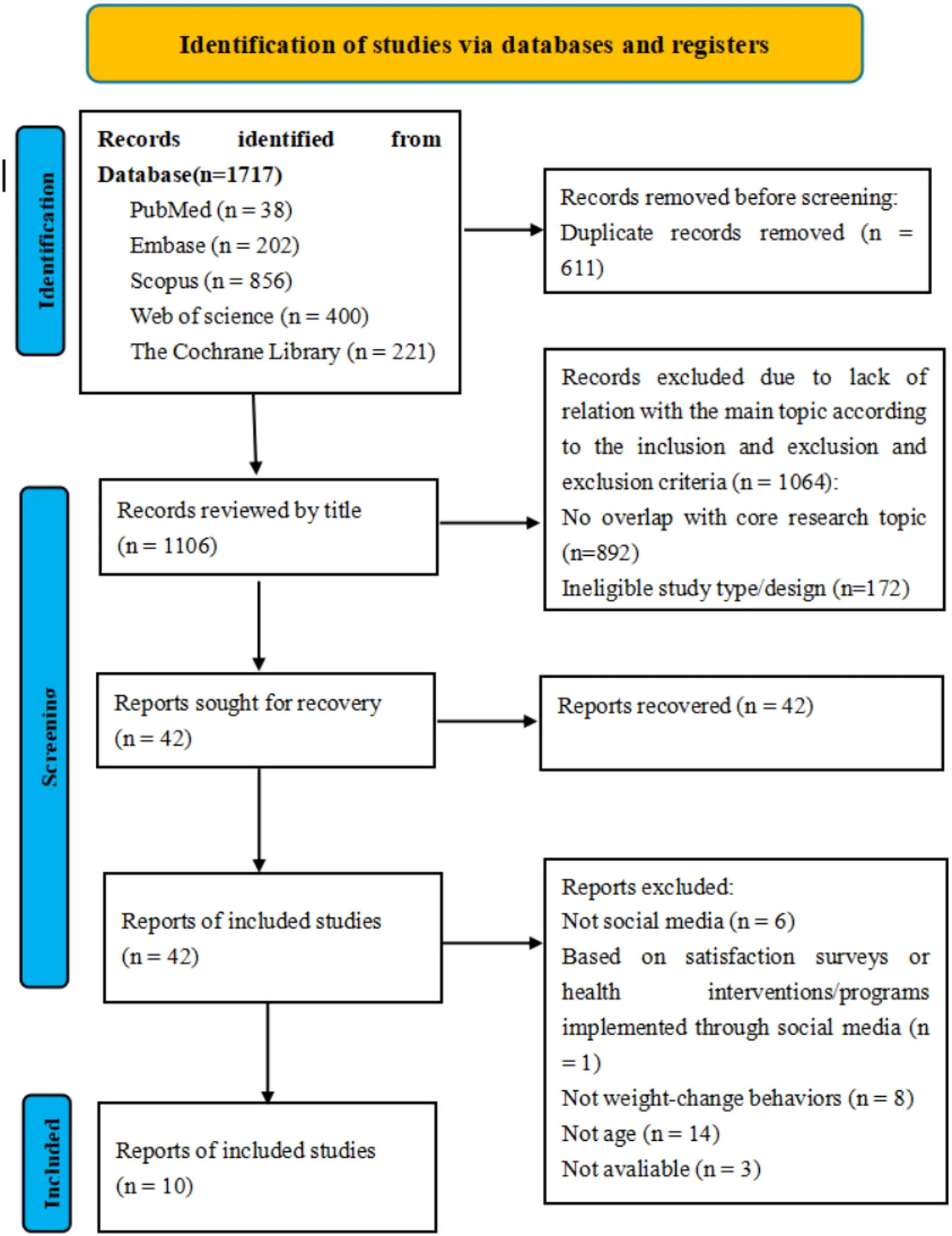
PRISMA-ScR flow diagram

The final selection of included studies was documented following the PRISMA-ScR flow diagram (Fig. 1).

### Data extraction

Three reviewers (J Zhou, L Xu, and Z Wang) independently extracted the data from a predesigned table in Microsoft Excel. Any disagreements between the two reviewers were resolved by consulting a third reviewer. Study characteristics, such as the first author of the reference, year, country, purpose, method, language, study population, sample size, data collection period and sampling method, are recorded in Table 1. Similarly, the main results of the studies on self-image and weight-change behaviors are presented in Tables 2 and 3.

**Table 1:** Results: study characteristics

Reference		Country, year	Objective	Design	Language	Study population	Sample size	Period	Selection
1	Feijoo et al. 2025 [28]	Spain, 2025	To explore how the perception of an aesthetically desirable body influences young people’s social opportunities and engagement in weight-loss behaviors, focusing on the mediating role of social media pressure.	Mixed-methods study	English	Spanish young people aged 12 to 17 years; healthcare professionals; fitness microinfluencers.	1082 adolescents; 6 experts.	Not explicitly specified, but inferred to be 2023–2024	Quantitative: Multistage stratified sampling across four geographical regions (NUTS classification), stratified by family socioeconomic status. Participants recruited through a panel service. Qualitative: Purposive sampling
2	Minadeo et al. 2022 [29]	USA, 2022	The purpose of this study was to identify themes present in popular food, weight, and body-related posts on TikTok.	Qualitative content analysis	English	Publicly available TikTok videos	1000 TikTok videos	September 2020	The participants were recruited from schools, youth centers and online community forums through a purposive sampling method.
3	Blanchard et al. 2023 [33]	Australia, Türkiye, Canada, China, Republic of Korea, United Kingdom, Indonesia, Italy, Japan, Norway, Spain, Philippines, 2023	To synthesize the literature on the associations between social media use and both adolescent mental health (specifically body image, self-esteem, stress, interpersonal relationships/loneliness, anxiety, and depressive symptoms) and diet.	Systematic Review	English	Adolescents aged 10–19 years	21 studies were included. The number of participants in the individual primary studies ranged from 62 to 244,250.	The literature search was conducted in January 2023. The included primary studies collected data between 2008 and 2022.	Participants were recruited through purposive sampling and snowball sampling. Schools, youth centers and community organizations in each country jointly participated in the recruitment process. The consent of parents and teenagers was obtained.
4	Suhag et al. 2024 [30]	India, 2024	To investigate how eating problems and young people’s body image are affected by social media, and to explore the link between social media usage and the development of binge eating disorder patterns and undesirable body image judgments.	Systematic review	English	Teenagers aged 13 to 18 who use social media.	35 studies included in qualitative and quantitative synthesis	Not explicitly stated, but the search was not time-limited.	Electronic databases (PubMed, Google Scholar), books, and grey literature were searched using keywords related to social media platforms and eating/body image disorders. Boolean operators were used to refine search results. PRISMA flow diagram was used for screening and selection.
5	Fiuza et al. 2023 [36]	USA, 2023	To examine the relationship between video-based platform use and body image, specifically the effects of brief diet and anti-diet TikTok videos on body image and eating concerns among young women.	Randomized Controlled Trial, RCT	English	U.S. female participants aged between 18 and 21 years who used social media.	Initially *n* = 421; after removing participants who failed the attention check, the final sample size was *n* = 398.	Data collection period not explicitly specified	Participants were recruited via a Qualtrics Survey Panel. They were randomly assigned to one of three conditions: (1) diet culture videos, (2) anti-diet videos, or (3) neutral videos. Participants who failed the attention check were removed from the analysis.
6	Lin et al. 2025 [37]	China, 2025	To examine the relationships between exposure to influencer marketing, dating app usage, body dissatisfaction (BD), and unhealthy weight-control behaviors (UWCBs) among young people.	Cross-sectional study	English	High-school students aged 15–18 years from urban and rural areas in Taiwan.	3121 valid questionnaires	2022 (during the COVID−19 pandemic)	Probability-proportionate-to-size sampling; 36 high schools from urban (Taipei, New Taipei, Kaohsiung) and rural (Pingtung, Yilan, Hualien) areas; students completed self-administered questionnaires in classrooms.
7	Murray et al. 2016 [31]	Canada, 2016	To examine whether body image concerns mediate the relationship between excessive time spent on social networking sites (SNS) and disordered eating behaviors among undergraduate students, and to explore potential gender differences in these relationships.	Cross-sectional study	English	Undergraduate students from Introductory Psychology classes at the University of Ottawa.	383 undergraduate students	Not explicitly specified	Convenience sample recruited from undergraduate courses; participants completed online or paper-based self-report questionnaires voluntarily or for course credit
8	Sanzari et al. 2023 [32]	USA, 2023	To compare patterns in social media use, body image disturbances, and disordered eating behaviors between two undergraduate student cohorts, and to examine if associations among social media use and disordered body image and eating behaviors are moderated by specific content consumed.	Cross-sectional with two time points (2015 and 2022) for cohort comparison	English	Undergraduate students at a large University in the northeastern United States.	2015 cohort: approximately 360 participants 2022 cohort: approximately 267 participants	Data collection occurred in two waves: 2015 and 2022.	Participants were undergraduate students recruited for course credit. The 2015 cohort participated via SurveyMonkey as part of a larger study, and the 2022 cohort was recruited remotely via email with a link to a Qualtrics survey.
9	Ganson et al. 2023 [35]	Australia, Canada, Chile, Mexico, United Kingdom, United States, 2023	To investigate whether screen time and social media use are associated with weight-change behaviors among an international sample of young people.	Cross-sectional study	English	Adolescents aged 10–17 years from six countries	12,031 participants	Data collection: November–December 2020 (web-based surveys)Data analysis: 2022.	Respondents were recruited through parents/guardians enrolled in the Nielsen Consumer Insights Global Panel and their partners’ panels. Email invitations with unique survey links were sent to adult panelists. Only one adolescent per household was invited. Quotas for age and sex were used in the UK and US.
10	Sampasa-Kanyinga et al. 2016 [34]	Canada, 2016	To investigate the associations between the use of SNSs and the perception of body weight and related behaviours among adolescent men and women.	Cross-sectional study	English	Canadian students in grades 7 to 12 (aged 11–19 years)	4468 students after excluding those with missing data on outcomes, explanatory variables, or covariates.	Data collection period: 2013	A two-stage cluster sample design involving a random selection of classes from within a random selection of schools (probability proportional to size) stratified by region and school type.

**Table 2:** Summary of findings: body image outcomes

Reference		Main results
1	Feijoo et al. 2025 [28]	Ideal body standard propagation: SM constructs physical appearance as “aesthetic capital,” linking a toned physique to social success and self-worth, thereby exerting persistent aesthetic pressure on adolescents. Mediating role of SM pressure: Perceived SM pressure partially mediates the association between aesthetic capital cognition, BD, and extreme eating behaviors, with a stronger effect observed in older females. Micro-influencer and algorithm effects: Fitness micro-influencers and algorithms reinforce fitness model-style ideal physiques even in health-oriented content, normalizing unrealistic standards and exacerbating BI anxiety.
2	Minadeo et al. 2022 [29]	Weight normativity dominance: Over 97% of TikTok weight-related videos convey weight normativity, implying health is confined to a specific weight range; creators are predominantly thin white individuals, lacking body diversity and reinforcing the thin ideal. Positive body image distortion: Approximately 21% of positive body image content focuses on post-weight-loss acceptance rather than non-standard body type affirmation, reflecting internalized weight bias. Moralization of body shape: SM frames body shape as a morality/health indicator, further distorting positive BI perceptions.
3	Blanchard et al. 2023 [33]	SM use correlates positively with BD and depressive symptoms; longer use duration and frequent appearance comparison are associated with poorer BI perception and lower self-esteem. Mediating role of psychological factors: BI, self-esteem, and anxiety mediate the relationship between SM exposure and eating behaviors. Platform and content effects: Multi-platform SM use induces ideal body internalization; following nutrition/beauty influencers triggers social appearance anxiety, with adverse effects exacerbated by poor marketing identification ability.
4	Suhag et al. 2024 [30]	Social comparison and BD: Exposure to curated ideal body images on SM exacerbates BD and low self-esteem (pronounced in young women) via upward social comparison. Thinspiration/fitspiration impact: Such content glorifies extreme thinness and muscularity, whose internalization induces BI distortion and persistent low body satisfaction. Algorithm and stigma effects: Algorithm-driven personalized content forms an information cocoon, reinforcing unrealistic standards; weight stigmatization further amplifies negative BI outcomes.
5	Fiuza et al. 2023 [36]	Diet video effects: Diet-related TikTok videos reduce body weight/shape satisfaction. Anti-diet video buffering effect: Anti-diet videos buffer negative BI by enhancing body appreciation. Moderating factors: These effects are moderated by thin-ideal internalization and SM rumination—high internalization/rumination correlates with more severe BI impairment, while low thin-ideal internalization predicts greater benefits from anti-diet content.
6	Lin et al. 2025 [37]	Dating app and marketing exposure: Dating app use and influencer weight management marketing exposure are positively associated with BD (pronounced in overweight/obese adolescents). Social pressure reinforcement: SM and dating apps jointly reinforce weight management social pressure, fueling weight-related internalized stigma. Unrealistic norm construction: Marketing and dating app content construct unrealistic norms of rapid weight loss and specific body types, further exacerbating BD.
7	Murray et al. 2016 [31]	SM use and body esteem: Increased SM use correlates with reduced appearance/body weight satisfaction and reinforced negative body perception. Mediating role of self-esteem: Weight/appearance self-esteem mediates the association between excessive SNS use and subsequent behavioral outcomes. Gender differences: Females’ appearance self-esteem and males’ weight self-esteem are differentially affected, with both genders exhibiting BI disturbances.
8	Sanzari et al. 2023 [32]	Content type vs. duration: Exposure to weight-loss content reduces body appreciation and increases fear of negative appearance evaluation, independent of SM usage duration. Algorithm-induced stigma: Platform algorithms deliver weight-loss content to form information cocoons, exacerbating weight stigma and self-denigration. Unrealistic goal setting: Trendy/rapid weight-loss regimens in SM content ignore individual nutritional/health needs, setting unrealistic weight-loss goals that impair BI perception.
9	Ganson et al. 2023 [35]	Regional and platform effects: Longer screen time and Twitter use increase weight dissatisfaction and subsequent weight gain/loss attempts, with regional differences in ideal body focus. Algorithm rabbit-hole effect: SM induces ideal body internalization via algorithm-driven content recommendations, whose rabbit-hole effect exacerbates body anxiety and BD.
10	Sampasa-Kanyinga et al. 2016 [34]	Gender-specific usage effects: Females with > 2 h/d of SM use exhibit higher BD, overweight misperception, and weight loss attempts; males with < 2 h/d of SM use have lower overweight perception risk. Interaction of SM use and gender: Females experience greater weight loss pressure with prolonged use, while males exhibit a buffering effect of short-term use on weight perception. Females’ body shape cognition: Heavy SM use in females induces overweight misperception and negative body shape cognitions.

**Table 3:** Summary of findings: weight-change behavior outcomes

Reference		Main results
1	Feijoo et al. 2025 [28]	Mediating role of SM pressure: Perceived muscularity-related pressure can reinforce the perception that muscularity is equivalent to social superiority, and this connection between cognition and weight change behavior is closely related to extreme dietary patterns (20.4% of people almost never eat, and 16.5% do not have regular meals). Social comparison and compulsive exercise: Upward social comparison triggers compulsive exercise urges. Micro-influencer misinformation: Fitness micro-influencers ignore body diversity and downplay individual differences (genetics/metabolism) when promoting extreme dieting/training, driving UWCBs.
2	Minadeo et al. 2022 [29]	Glorification of rapid weight loss: TikTok content glorifies and normalizes rapid weight loss, framing it as a willpower issue while ignoring individual differences/sustainability and concealing weight regain/repeated dieting. Food moralization: Food is labeled as beneficial or harmful, and is transformed into a tool for weight control, thereby increasing the risk of developing diabetes. Unscientific nutrition advice: Non-professional creators disseminate unsubstantiated nutritional advice (e.g., fad diets, liquid cleanses), misleading adolescents into unhealthy weight management practices.
3	Blanchard et al. 2023 [33]	Thin-ideal account following: Following thin-ideal SM accounts increases BD risk and subsequent binge eating/excessive dieting. Influencer-driven anxiety: Over 50% of adolescents follow fitness/nutrition bloggers, whose content exacerbates social appearance anxiety/emotional eating and drives extreme weight control. Dietary pattern impacts: SM use correlates with internet-famous food preference, fast food consumption, and impaired hunger/satiety perception. Influencer-driven social appearance anxiety triggers emotional eating and drives extreme weight control behaviors.
4	Suhag et al. 2024 [30]	Unscientific weight-loss dissemination: SM disseminates unscientific dietary information (folk remedies and fad diets) and extreme weight-loss approaches via influencers, inducing UWCBs. Social comparison and self-objectification: These processes trigger unregulated dieting, weight-loss medication misuse, and excessive exercise. Usage duration correlation: SM usage duration correlates positively with UWCB engagement, with stronger effects observed in females.
5	Fiuza et al. 2023 [36]	Diet culture video effects: Diet culture videos significantly increase dietary restriction intentions and abnormal exercise urges, normalizing unhealthy behaviors via extreme dieting/exercise promotion. Anti-diet video benefits: Anti-diet videos enhance intuitive eating and reduce pathological weight concern. Reinforcement by rumination: Thin-ideal internalization increases extreme weight-change behavior risk, and video content rumination strengthens dietary restriction impulses.
6	Lin et al. 2025 [37]	Influencer marketing and UWCBs: High influencer weight control marketing exposure correlates positively with UWCBs; influencer weight-loss products often involve fraudulent advertisements, misleading adolescents to adopt ineffective/harmful methods. BD as a vulnerability factor: BD increases vulnerability to influencer marketing, inducing extreme weight loss methods (dieting, binge eating, fasting, self-induced vomiting).
7	Murray et al. 2016 [31]	Self-esteem impairment and eating behaviors: Excessive SM use impairs appearance and weight self-esteem, inducing restrictive eating in both genders and additional emotional eating in females. Gender-specific aesthetic norms: SM propagates gender-specific aesthetic norms (thin ideal for females, muscular ideal for males), driving dietary restriction in females and extreme training in males, leading to DE in both. Unscientific trend promotion: SM-promoted unscientific weight-loss trends induce unhealthy weight modification approaches.
8	Sanzari et al. 2023 [32]	Extreme behavior exacerbation: SM content exacerbates extreme dietary and weight-control behaviors (binge eating, vomiting, laxative abuse), with impact magnitude correlating with exposure duration. Gender-specific behaviors: SM impairs body esteem, strengthening restrictive dieting tendency in females and excessive exercise in males. Limited positive content effect: Body-positive and neutral content fails to offset these negative effects.
9	Ganson et al. 2023 [35]	Ideal-driven weight attempts: SM use correlates with weight loss/gain/maintenance attempts (driven by lean and muscular ideal pursuit), increasing dieting and excessive exercise. Extreme practice propagation: SM weight-loss content directly fuels extreme practices (vomiting, laxative abuse), with such behaviors showing an upward trend in recent years.
10	Sampasa-Kanyinga et al. 2016 [34]	Cross-country and platform correlation: SM use correlates positively with weight-loss dieting across countries and platforms. Gender differences in behaviors: Females exhibit more weight loss attempts, while males engage in more weight gain/extreme training for muscularity. Dose-response relationship: SM drives developmentally inappropriate weight-control strategies and increases extreme behavior risk, with a dose-response relationship between content exposure and risk.

## Results

### Search and selection results

The preliminary search of all the databases analysed yielded 1717 results.

According to the inclusion criteria, only 42 articles that met the requirements were screened in after the titles and abstracts were read. After a full-text screening, 32 articles that did not meet the review objectives were ultimately excluded: 1 article was related to health projects conducted through social media, 14 articles did not meet the age limit of 10–24 years, 3 articles could not be obtained, 6 articles did not explore the relationship between social media and body image, and 8 articles did not analyze the impact of social media on weight. Finally, 10 articles met the inclusion criteria and were selected for the scoping review.

The PRISMA-ScR flow diagram (Fig. 1) represents the entire process.

### Characteristics of the articles

The studies included in this review were published between 2016 and 2025. They mainly adopted cross-sectional studies, qualitative content analysis, and systematic reviews as their design. A few studies involved mixed-method research, experimental studies, and two-time-point cohort comparison studies. Most of the studies focused on platforms such as TikTok, Instagram, Facebook, and Snapchat, which are the social media most commonly used in research on body image and weight-change behaviors among young people. Some studies have focused on platforms related to appearance display, such as dating apps. In terms of geographical distribution, the research coverage was extensive and included Western countries such as the United States, Canada, Australia, and the United Kingdom, as well as non-Western countries such as China, India, Chile, and Mexico.

Notably, two of the included studies were systematic reviews, which contributed unique geographical and synthetic evidence that could not be obtained from individual primary studies. Blanchard et al. (2023) synthesized evidence from 21 studies across 12 countries, providing the most comprehensive cross-cultural comparison available. Suhag et al. (2024) focused exclusively on South Asian adolescents, offering insights into cultural variations in the relationship between social media content and weight-change behaviors that were absent from the Western-dominated primary study literature. No overlap was found between the primary studies included in these two reviews and the other eight primary studies in our sample, as detailed in the Methods section.

Table 1 summarizes the main characteristics of the selected studies, and Tables 2 and 3 summarize the main results of the selected studies.

### Direct effects of SM content on body image perception

The scoping review indicates that social media exerts a significant impact on young people’s perception of body image.

Social media disseminates fitness, nutrition, and weight-loss content predominantly featuring lean or muscular body types, which fails to reflect the natural body diversity of young people [28, 29]. The internalization of these idealized standards is driven by peer interactions, algorithmic recommendations, and the lack of diverse body representations in SM weight-loss content [29, 30]. Appearance comparison facilitated by SM emerges as a core predictor of BD [30]; the widespread exposure to appearance-related content and availability of image-editing tools on SM platforms leads to continuous exposure to unrealistic and unattainable body images, perpetually triggering upward social comparisons (i.e., perceiving others as more desirable) and subsequent self-objectification [31, 32].

The frequency of exposure to others’ appearance content and perceived attractiveness advantages directly induce negative emotions and BD in young people, independent of behaviors such as posting selfies or receiving likes [31–33]. Furthermore, exposure to appearance-focused SM content has a more pronounced impact on BI than overall SM usage frequency [32], and multi-platform SM use increases the likelihood of idealized standard internalization [32, 33]. Social media fitness influencers play a pivotal role in driving the internalization of thin and muscular ideals, exacerbating aesthetic pressure on young people and reinforcing narrow body standards [28, 30]; even influencers aiming to promote health-oriented content are driven by SM algorithms to prioritize physique-focused content, which normalizes unrealistic body standards and entrenches BI anxiety [28].

The frequency and duration of SM usage are associated with BD in both genders, with a more pronounced effect observed in females [31, 33]. Females are more susceptible to the thin ideal and tend to internalize standards of a toned, curvaceous physique, whereas males are more likely to internalize the muscular ideal [33, 35]. Anti-diet and body-positive SM content (e.g., TikTok body acceptance videos) exhibits a buffering effect on negative BI, but this effect is significantly moderated by the degree of ideal standard internalization [36]; young people with low thin-ideal internalization derive greater benefits from such content, with improved body satisfaction and self-evaluation [36]. Parental supervision and regular physical activity were identified as consistent protective factors that reduce BD and improve emotional well-being in young people [35].

### Mediating role of BD in SM-induced weight-change behaviors

This review systematically summarizes the direct and indirect impacts of social media on weight-change behaviors among young people, while clarifying the exposure-related opportunities and potential risks in this field.

Social media disseminates gender-differentiated idealized body standards and constructs an aesthetic hierarchy centered on physical attractiveness [28, 35], driving the internalization of such standards and subsequent BD. Widespread SM use further motivates active weight-loss behaviors [30, 35], and TikTok weight-loss content as well as influencer marketing of weight-reduction products directly induce dietary changes and product purchases by glorifying weight-loss outcomes and reinforcing weight-related normative perceptions [29, 37]. Prolonged SM use impairs BI, reduces appearance-related self-esteem, and triggers restrictive eating, disregard for hunger/satiety cues, and frequent fast food consumption [31, 33]. Daily SM exposure is associated with weight gain, weight loss, and dieting behaviors, manifesting as elevated BD and increased desire for weight loss [34, 35], with SM content type exerting a more prominent impact on weight behaviors than usage duration or platform diversity [32].

Peer appearance comparison, thin-ideal internalization, and social appearance anxiety induced by SM exposure significantly increase the risk of BD and eating disorders [30, 33, 36], which further predict behaviors such as dieting, fasting, and reliance on weight-loss products—with higher risks among overweight or obese young people [28, 37]. Algorithmic personalized weight-loss content, “thinspiration/fitspiration” trends, and coercive discourses attributing weight-loss success solely to individual effort collectively drive extreme weight-change behaviors [29]. Additionally, selfie-related behaviors, frequent use of specific SM platforms, exposure to influencer weight-loss marketing, and dating app use are significantly associated with eating disorder symptoms and UWCBs (e.g., fasting, laxative abuse, self-induced vomiting) [32, 33, 37].

Distinct gender differences in weight-change behaviors were observed: females are more susceptible to lean-ideal pressure and tend to pursue weight loss, whereas males are driven by the muscular ideal to engage in extreme exercise [28, 34]. Notably, weight- and appearance-related self-esteem mediates the association between excessive SM use and restrictive dieting across both genders [31]. While body-positive SM content can stimulate physical activity enthusiasm and guide young people to prioritize overall health over extreme body shapes [30], such positive effects are negligible in the context of mainstream negative SM content. Furthermore, unregulated and evidence-lacking weight-loss methods (e.g., unproven high-protein low-calorie diets, liquid “cleanses”, inappropriate adolescent intermittent fasting) are widely advocated on SM due to insufficient regulatory oversight, further exacerbating health risks for young people [29, 36].

#### Quality of evidence

Two reviewers (J Zhou and Z Wang) independently assessed the methodological quality of the included studies for descriptive purposes only. The assessment tools included the Standards for Reporting Qualitative Research (SRQR) statement [38], the Consolidated Standards of Reporting Trials (CONSORT) statement [39] for randomized controlled trials, the Mixed Methods Appraisal Tool (MMAT) statement [40] for mixed-methods studies, the Strengthening the Reporting of Observational Studies in Epidemiology (STROBE) statement [41] for epidemiological observational studies, and the Preferred Reporting Items for Systematic Reviews and Meta-Analyses (PRISMA) statement [42] for systematic reviews. Most included studies were rated as low quality, primarily due to cross-sectional design and self-reported data. However, core findings were consistent across moderate- and low-quality studies, reducing the risk of bias-induced distortion. No formal heterogeneity statistical analysis or sensitivity analysis was performed in this paper. Heterogeneity was presented only through descriptive integration. No formal risk of bias assessment was conducted, which is consistent with the methodological recommendations for scoping reviews [27]. The quality of evidence for the articles is detailed in Table 4.

## Discussion

### Main findings

This scoping review focuses on exploring the correlation between young people’s.

exposure to social media content and their body image and the development of weight-change behaviors. The literature indicates that social media may play a significant role in influencing young people’s body image and the formation of weight-change behaviors.

### Influence of exposure to social media on body image perception

The included studies confirm that SM’s impact on young people’s BI perception is mediated by multiple interrelated mechanisms, consistent with social comparison theory and self-objectification theory. These results align closely with prior systematic reviews and meta-analyses that have established social comparison as a central mediator between SM use and body dissatisfaction [23, 43]. Unlike traditional media, SM algorithms deliver personalized, idealized body content that forms an “information cocoon” [30], reinforcing unrealistic body standards and exacerbating BI anxiety and self-denigration [35, 37].

Second, Dane et al. [24] noted in their review that fitness micro-influencers serve as critical conduits for the dissemination of idealized body standards. However, this scoping review reveals that even content posted by micro-influencers claiming to promote healthy lifestyles is driven by algorithmic mechanisms to prioritize physique-focused material, thereby normalizing narrow body standards and exacerbating aesthetic pressure [28]. This finding elucidates why the prevalence of body image anxiety among adolescents has continued to rise in recent years, despite the growing proportion of so-called “health-focused” content on social media.

Thirdly, content type (e.g., weight-normative messaging, and algorithm-driven thin-ideal posts) exerts a more significant and independent influence on body image than the total duration or frequency of social media use [29, 32, 36]. This finding supports the viewpoint put forward by Cohen et al. [44] that interventions aimed merely at reducing screen time may be less effective than strategies based on content regulation.

Notably, the protective effects of anti-diet and body-positive SM content are conditional and limited: such content only benefits young people with low thin-ideal internalization [36], and many so-called “body-positive” SM posts still conform to mainstream aesthetic standards (e.g., celebrating weight loss rather than accepting non-standard body types) [29, 32], negating their protective value. Parental supervision and physical activity were identified as the only consistent protective factors against negative BI [35], highlighting the role of offline factors in mitigating SM’s adverse effects.

### The impact of social media usage on weight-change behaviors

The findings of this literature review highlight the dual effects of social media on the promotion of weight-change behaviors among young people.

Notably, the type of social media content constitutes the primary factor influencing weight-control behaviors. Prolonged exposure to weight-loss/fitness-related content leads to behaviors such as restrictive eating, excessive exercise, and binge eating, and this impact is independent of the overall duration of social media use [32]. Algorithmically curated content, particularly thinspiration and fitspiration imagery, establishes personalized feedback loops that perpetually reinforce idealized body standards, rendering passive exposure to such targeted content significantly more psychologically impactful than general, non-targeted platform usage [30, 32]. Video platforms such as TikTok are inherently characterized by high interactivity and immersiveness, as the synergistic integration of dynamic visuals, on-screen text, and background music elicits more pronounced affective responses compared to static imagery [36]. Cohen et al. corroborated this perspective by demonstrating a dose-response relationship between targeted engagement with appearance- or weight-related content and unhealthy weight control behaviors [44]. This finding challenges the common assumption that “time spent on SM” is the key risk factor, highlighting the need to focus on content regulation rather than just usage time.

Second, gender differences in weight-change behaviors are rooted in the internalization of distinct idealized body standards: Females exhibited greater engagement in dietary restriction and laxative misuse, whereas males demonstrated higher rates of excessive exercise and weight-gain attempts—a pattern consistent with the findings reported in the meta-analysis by Bonfanti et al. [23].Notably, weight- and appearance-related self-esteem mediates the association between SM use and restrictive dieting in both genders [31], indicating that males are not immune to SM-induced UWCBs.

Social media also drives UWCBs through two additional mechanisms: first, the dissemination of unscientific weight-management information (e.g., fad diets, weight-loss folk remedies) by non-professional SM creators [29] misleads young people into adopting unregulated weight-loss methods; second, influencer marketing of weight-loss products on SM and dating apps features fraudulent advertisements and unrealistic weight-loss claims [37], further driving UWCBs among young people with BD.

### Potential mediating factors such as social comparison and the internalization of thin ideals

This study found that there are multiple pathways of association between social media use and body dissatisfaction as well as weight-change behaviors.

Social appearance comparison enabled by SM is the primary mediator between SM use and BD [30, 31]; Previous research has demonstrated that appearance comparison increases the risk of BD by 37% [30]. This mediating effect is universal across genders and SM platforms, and is amplified by algorithmic information cocoons [30].

The internalization of thin/muscular ideals moderates the association between SM content exposure and BD [36]; young people with high thin-ideal internalization are more susceptible to the adverse effects of diet-related SM content, and even body-positive content fails to mitigate their BD [36]. Social media-related rumination further exacerbates this effect, leading to reduced body appreciation and increased restrictive eating [36].

Self-esteem, especially in terms of body weight and appearance, can regulate the relationship between excessive use of social media and disordered eating behaviors [31]. Females are more prone to emotional eating due to reduced appearance self-esteem, while both males and females engage in restrictive dieting as a result of decreased weight/appearance self-esteem [31, 33]. This indicates that males and females face similar psychological risks induced by social media exposure, despite differences in their behavioral outcomes.

### Limitations and recommendations for future studies

Despite the value of the findings, there are inherent limitations.

First, the methodological quality of the included body of evidence was generally low. The majority of studies were assessed as low quality, with only three attaining a moderate quality rating. Notable methodological limitations included inadequate control of key confounding variables [31, 33, 35, 37] and the use of overly simplistic, insufficiently validated measurement instruments for complex constructs such as social media content exposure and body dissatisfaction [31, 34, 37]. Collectively, these limitations undermine the reliability and generalizability of the synthesized findings, warranting cautious interpretation of the conclusions drawn.

Most of the included studies adopted a cross-sectional design [31, 32, 34, 35, 37], which makes it impossible to determine the generalizability and causality of the research results. Most studies relied on self-reported data, which poses a risk of recall bias and social desirability bias. Additionally, the study subjects were young people, and the impact on a broader age range remains unclear. Most of the included studies enrolled predominantly female participants, which constrains the generalizability of gender-based comparisons and underscores the need for more gender-balanced samples in future research.

Future research should prioritize longitudinal designs or experimental methods to further investigate the causal relationships between social media use and body image and weight-change behaviors. It is recommended that the cultural and geographical scope of the study subjects be expanded, especially by conducting more research in regions such as Asia, Africa, and Latin America, to increase the generalizability of the conclusions. Moreover, studies should focus on the differences among various gender, sexual, racial, and socioeconomic background groups; identify high-risk groups; and develop targeted intervention strategies.

**Table 4:** Quality of evidence

Reference		Design	Quality assessment tool	Quality of evidence
1	Feijoo et al. 2025 [28]	Mixed-methods study	MMAT	Moderate. Although the research performed well in terms of mixed-methods design, integration, and interpretation, the nonresponse bias in the quantitative part and the limitations of self-reported data reduced the reliability of the evidence. Additionally, the sample was limited to Spanish young people, which restricted the generalizability of the results in other cultural contexts.
2	Minadeo et al. 2022 [29]	Qualitative study	SRQR	Moderate. The evidence is affected by measurement bias, insufficient control of confounding factors, and the use of a single credibility-enhancing technique. The neglect of nonhighly viewed content, algorithmic factors, and the actual user contact scenarios has limited the credibility, transferability, and applicability of the research results in other contexts.
3	Blanchard et al. 2023 [33]	Systematic review	PRISMA	Moderate. This evidence has deficiencies in aspects such as bias risk control, heterogeneity handling, evidence quality assessment, and data sharing. These limitations affect the credibility of the research results and the policy transformation capability, especially in terms of the practicality for guiding clinical practice.
4	Suhag et al. 2024 [30]	Systematic review	PRISMA	Low. Core limitations: unclear missing data handling, lack of heterogeneity/sensitivity analysis, and narrative synthesis without quantitative pooling; secondary limitations: incomplete reporting of study characteristics and selective reporting risk.
5	Fiuza et al. 2023 [36]	Randomized Controlled Trial, RCT	CONSORT	Low. This experimental design provides a certain methodological reliability. However, the use of mean values for incomplete data without verifying their rationality, the absence of trial registration and complete protocols, the failure to report adverse events, and the risk of selective reporting of subgroup analyses all reduce the quality of the evidence.
6	Lin et al. 2025 [37]	Cross-sectional study	STROBE	Low. The specific handling strategies for missing data were not specified, the measurement of key variables lacked validation of reliability and validity, no sensitivity analysis was conducted, and the reasons for selecting confounding factors were insufficient. There is a potential risk of selective reporting, which reduces the quality of the evidence.
7	Murray et al. 2016 [31]	Cross-sectional study	STROBE	Low. The basis for sample size calculation, strategies for handling missing data, and sensitivity analysis were not specified. The validity of the SNS excessive use scale was not adequately verified, and there is a potential risk of selective reporting, which reduces the quality of the evidence.
8	Sanzari et al. 2023 [32]	Cross-sectional cohort comparison	STROBE	Low. This research design provides a certain level of methodological reliability. However, it does not specify the basis for sample size calculation, only reports the proportion of missing data without clearly stating the handling strategy, does not conduct sensitivity analysis to verify the stability of the results, the measurement dimensions of social media content are single, and there is a potential risk of selective reporting, which reduces the quality of the evidence.
9	Ganson et al. 2023 [35]	Cross-sectional study	STROBE	Low. The evidence was affected by selection bias, measurement bias, and insufficient control of confounding factors. The lack of strict sampling representativeness and comprehensive consideration of confounding factors limited the practicality of the research results in guiding the establishment of screen usage norms and weight management interventions for young people in different regions.
10	Sampasa-Kanyinga et al. 2016 [34]	Cross-sectional study	STROBE	Low. This research design provides a certain level of methodological reliability. However, it does not specify the basis for sample size calculation, the reasonableness of the exclusion method used for missing data has not been verified, no sensitivity analysis was conducted, the measurement dimensions of SNS were not detailed, there is a potential risk of selective reporting, and the quality of the evidence has been reduced.

### Analysis of the quality of evidence

A methodological quality analysis of the studies included in this review identified key limitations that may compromise the robustness, applicability, and generalizability of the research findings. The majority of the studies were rated as low quality, with only three studies attaining a moderate quality level; however, their inherent flaws still undermined the reliability of the conclusions drawn. Core limitations were manifested in several aspects, including inadequate sample representativeness, insufficient control of confounding factors, oversimplified measurement tools, and the potential risk of selective reporting.

The most common flaw is the lack of sample representativeness. Research based on platforms such as TikTok overly relies on highly exposed content. For example, Minadeo et al. [29] analysed only high-view tag videos, neglecting low-exposure content, which makes the sample fail to represent the platform’s overall picture. Many studies are confined to specific geographical regions, cultural backgrounds, or demographics, limiting the diversity of participants and the external validity of research findings, making it difficult to generalize the conclusions to nondigital groups, private school students, or those in low- and middle-income countries.

Qualitative and observational studies are generally plagued by content analysis bias. For example, Sanzari et al. [32] simplified social media content exposure to a binary variable of “whether seeking”, which fails to quantify dose effects. Many studies rely on single items or inadequately validated tools to measure complex core constructs, introducing severe measurement bias and weakening the comparability of results. For example, Lin et al. [37] used a single question to assess body dissatisfaction, Sampasa-Kanyinga et al. [34] classified SNS behavior only by usage time, and Murray et al. [31] used an adapted scale without validated cross-population validity to assess SNS overuse. Such simplifications significantly reduce the accuracy of the data.

Although cross-sectional studies form the bulk of the evidence, they generally fail to adequately measure and adjust for key confounding variables such as mental health history, family environment, media literacy, and socioeconomic status [31, 33, 35, 37], which may lead to misleading conclusions about associations.Only a few studies reached a moderate quality level, yet there were obvious limitations, such as improper handling of missing data and failure to report adverse events in RCTs [36], inclusion of high-bias-risk studies and inadequate handling of heterogeneity in systematic reviews [33], and a single perspective in the qualitative component of mixed-methods studies [28]. This highlights the overall lack of methodological rigor in this field.

In summary, although the included studies have provided initial and important insights into the complex relationship between social media and young people’ body image and weight-change behaviors, the overall conclusion is difficult to generalize to nonactive users, culturally diverse groups, and real-world scenarios because of the overconcentration of samples on specific platform algorithm-filtered users, educational institutions, or high-income countries. Despite the moderate quality of the studies providing preliminary insights, methodological flaws require a highly cautious attitude towards the results.

This review emphasizes that future research should adopt methodologically rigorous designs, enhance sample representativeness, strictly control confounding factors, and maintain transparency in result reporting to strengthen the existing empirical foundation in this emerging field.

### Implications for clinical practice and public health

This review emphasizes the necessity of focusing on the impacts of social media on body image and weight-change behaviors among young people. As a primary channel for young people to obtain information and establish social connections, social media platforms can exert positive effects by promoting diverse body image content that aligns with the physical and mental development characteristics of this population, while avoiding the excessive promotion of a narrow aesthetic standard that glorifies extreme thinness or muscularity.

Given the finding of the present study that over 97% of weight-related TikTok videos convey weight-normative messaging [29], the platform may prioritize adding ‘idealized body image reminder’ labels to content associated with body weight management and dieting [45]. Simultaneously, it could remove the display of like counts, add disclaimers pertaining to scientific weight loss, and strengthen protective measures against content involving body weight discrimination, thereby fostering a safe digital environment for young people [45]. Health communicators should continuously integrate media literacy and digital literacy into intervention programs targeting young people, helping them recognize the potential impacts of media on their emotions and experiences. Furthermore, policymakers and the technology industry need to establish relevant mechanisms to alleviate the burden of responsibility on young people and mitigate the negative effects of harmful content [46].

In addition, the implementation of targeted media literacy enhancement programs is crucial, and the cultivation of positive self-perception and body acceptance is equally indispensable. The widespread lack of formal media literacy education may lead to a decline in the level of critical engagement with social media among young people [47]. Faced with the heterogeneous quality of fitness information available on social media, guiding young people to seek professional advice from dietitians and physicians represents a key step in preventing physical harm and ensuring their healthy development [28].

## Conclusion

This review indicates that social media exerts a significant impact on young people’s body image cognition and weight-change behaviors. While these platforms can provide vital support and information, the over-proliferation of content promoting idealized aesthetic standards may exacerbate body dissatisfaction; in the long run, it may even induce young people to adopt unhealthy weight management strategies. To mitigate these adverse effects, we advocate the promotion of inclusive and evidence-based content, coupled with the enhancement of media literacy education and emotional support interventions, with a primary focus on safeguarding the overall health and well-being of young people.

Furthermore, it is recommended that healthcare professionals and public health institutions actively collaborate with digital content creators and social media platforms to disseminate validated health information and launch joint initiatives. Study findings demonstrate that cooperative internet celebrities can jointly advance programs aimed at improving users’ social media literacy [48]. Such activities can be implemented in the form of lectures and group discussions held in schools and universities, to educate clinicians, educators, and parents about the risks and opportunities associated with social media, including the consumption of internet celebrity content [7]. Such collaborations facilitate the dissemination of accurate health information, combat misinformation, and enhance the visibility of diverse and authentic body images, thereby better reflecting the real-life experiences of young people.

## Supplementary Information

Supplementary Material 1.

## Data Availability

No datasets were generated or analysed during the current study.

## Abbreviations

SM: Social Media
BI: Body Image
PIO: Participants, interventions, and outcomes
BD: Body Dissatisfaction
MMAT: Mixed Methods Appraisal Tool
CONSORT: Consolidated Standards of Reporting Trials
WOS: Web of Science
STROBE: Strengthening the Reporting of Observational Studies in Epidemiology
SNS: Social Networking Sites
SRQR: Standards for Reporting Qualitative Research
UWCBs: Unhealthy Weight-Control Behaviors
NUTS: Nomenclature of Territorial Units for Statistics
PRISMA-ScR: Preferred Reporting Items for Systematic Reviews and Meta-Analyses Extension for Scoping Reviews
