# Supplementary material for: The impact of social media on body image and weight-change behaviors among young people: a systematic scoping review
Source: J Eat Disord. 2026 Aug 8;14:267. doi: 10.1186/s40337-026-01717-4 (PMC13761181; doi:10.1186/s40337-026-01717-4)
Supplement: Supplementary file 1 [file 40337_2026_1717_MOESM1_ESM.docx]

Table S1. Generic search terms.

| **CONCEPT** | **SEARSH TERMS** |
| --- | --- |
| **Body Image** | "Body Image" OR "Body Representation" OR "body perception" OR "body surveillance" OR "body shame" OR "body comparison" OR "body checking" OR "body image avoidance" OR "body image disturbance" OR "body dissatisfaction" OR "body negativity" OR "body positivity" OR "body acceptance" OR "body appreciation" OR "body satisfaction" OR "thin ideal" OR "weight stigma" OR "body image anxiety" |
| **social media** | "social media" OR "social networking site" OR "Instagram" OR "TikTok" OR "Snapchat" OR "Facebook" OR "Twitter" OR "X" OR "Pinterest" OR "YouTube" OR "social network" OR "tweet*" OR "Tumblr" OR "LinkedIn" OR "blog*" OR "social media content" OR "dating app*" OR "fitspiration" OR "thinspiration"* |
| **young adult** | "youth*" OR "adolescent*" OR "young adult*" OR "teen*" OR "young people" |
| **body weight** | "body weight" OR"body weight change*" OR "body weight management* "OR "body weight control* "OR" weight loss*" OR "weight loss maintenance" OR "weight regain*" OR "weight loss behaviors"OR "unhealthy weight-control behavior*" |

Literature search (PubMed-specific)

DATEBASE:Pubmed

SEARCH STRATEGY:

("Body Image" OR "Body Representation" OR "body perception" OR "body surveillance" OR "body shame" OR "body comparison" OR "body checking" OR "body image avoidance" OR "body image disturbance" OR "body dissatisfaction" OR "body negativity" OR "body positivity" OR "body acceptance" OR "body appreciation" OR "body satisfaction" OR "thin ideal" OR "weight stigma" OR "body image anxiety") AND ("social media" OR "social networking site" OR "Instagram" OR "TikTok" OR "Snapchat" OR "Facebook" OR "Twitter" OR "X" OR "Pinterest" OR "YouTube" OR "social network" OR tweet* OR "Tumblr" OR "LinkedIn" OR blog* OR "social media content" OR "dating app"* OR "fitspiration" OR "thinspiration") AND (youth OR adolescent* OR "young adult"* OR teen* OR "young people") AND ("body weight" OR "body weight change"* OR "body weight management"* OR "body weight control"* OR "weight loss"* OR "weight loss maintenance" OR "weight regain"* OR "weight loss behaviors" OR "unhealthy weight-control behavior"*)

SEARCH FILTERS:in the last 10 years

RESULTS:38
